# Homeostasis of Molybdenum, Xanthine Oxidase and Aldehyde Oxidase Activity Levels in the Livers of Patients with End-Stage Liver Failure and Healthy Controls

**DOI:** 10.3390/ijms27114998

**Published:** 2026-05-31

**Authors:** Jakub Piotr Adamus, Aleksandra Wyczałkowska-Tomasik, Anna Ruszczyńska, Ewa Bulska, Zuzanna Ząbek, Karolina Pastwa, Dariusz Sołdacki, Leszek Pączek

**Affiliations:** 1 Department of Clinical Immunology, Medical University of Warsaw, 59 Nowogrodzka Street, 02-006 Warsaw, Poland; jadamus.md@gmail.com (J.P.A.); leszek.paczek@wum.edu.pl (L.P.); 2 Clinical Immunology Student Scientific Association, Medical University of Warsaw, 59 Nowogrodzka Street, 02-006 Warsaw, Poland; zuzanna.zabek2@gmail.com (Z.Z.);; 3 University of Warsaw, Faculty of Chemistry, Biological and Chemical Research Centre, 101 Zwirki i Wigury Street, 02-089 Warsaw, Poland; ebulska@chem.uw.edu.pl; 4 Longevity Center Europe, 9 Belwederska Street, 00-761 Warsaw, Poland; dariusz.soldacki@gmail.com; 5 Institute of Biochemistry and Biophysics, Polish Academy of Sciences, 5A Pawinskiego Street, 02-106 Warsaw, Poland

**Keywords:** liver transplantation, molybdenum, xanthine oxidase, aldehyde oxidase, end-stage liver failure, oxidation-reduction

## Abstract

Xanthine oxidase (XO) and aldehyde oxidase (AO) are molybdenum-dependent flavoenzymes involved in purine catabolism and hepatic xenobiotic metabolism. This study aimed to assess XO and AO activities and molybdenum (Mo) content in liver tissue from liver graft donors and recipients with end-stage liver failure (ESLF). Liver tissue samples were obtained from 10 liver donors (LD; control group) and 30 patients with ESLF undergoing liver transplantation. The ESLF cohort was further divided by primary liver disease: hepatitis B or C viral infection (HBV/HCV), alcohol-associated liver disease (ALD), primary sclerosing cholangitis (PSC), Wilson’s disease (WD), and primary biliary cholangitis (PBC). Enzymatic activity was determined by spectrofluorometry, and hepatic Mo content was measured by inductively coupled plasma mass spectrometry. Compared with LD controls, ESLF recipients showed a significant decrease in both hepatic XO activity and Mo content. At the subgroup level, XO activity was significantly reduced in ALD, PSC, and PBC, whereas HBV/HCV and WD showed lower but non-significant values. Mo content was significantly reduced in the overall ESLF cohort and in the WD subgroup, although disease-specific variability was observed, including relatively high Mo content in PSC. AO activity was assessed in a subset of samples and did not differ significantly between donor and recipient liver tissues. Correlation analyses did not demonstrate a statistically significant association between hepatic Mo content and XO activity, suggesting that total tissue Mo content may not directly reflect Mo-dependent enzymatic activity. These findings indicate that ESLF is associated with altered hepatic trace-element composition, including Mo, and with disturbances in Mo-dependent enzymatic activity. Future studies should combine liver tissue analysis with matched serum and portal blood samples to better characterise hepatic Mo metabolism and the systemic biological effects of trace elements.

## 1. Introduction

Molybdenum (Mo) is an essential trace element whose biological activity in humans depends primarily on its incorporation into the molybdenum cofactor (MoCo), rather than on free ionic Mo. MoCo is a pyranopterin-based organometallic cofactor in which the Mo centre is coordinated by the cis-dithiolene group of a molybdopterin (MPT), a tricyclic pyranopterin ligand, thereby forming the catalytic centre of human molybdoenzymes [1,2]. Four MoCo-dependent enzymes are currently recognised in humans: xanthine oxidase (XO), aldehyde oxidase (AO), sulphite oxidase (SOX), and the mitochondrial amidoxime-reducing components mARC1 and mARC2 [1,3]. These enzymes link Mo availability and MoCo-dependent catalysis to purine catabolism, aldehyde and xenobiotic metabolism, sulphite detoxification, and mitochondrial reductive metabolism [1,3,4]. Among them, SOX is particularly critical for systemic Mo-dependent homeostasis because it catalyses the oxidation of sulphite to sulphate in sulphur amino acid metabolism; accordingly, loss of SOX activity is the major biochemical driver of the severe neurological phenotype observed in MoCo deficiency (MoCD) [4,5].

Xanthine oxidase is an important molybdenum-containing flavoenzyme that plays a vital role in purine metabolism [6,7]. It catalyses the two-step hydroxylation of hypoxanthine to xanthine and then to uric acid [6,7]. Structurally, XO is a dimer, with each monomer containing three key components: MoCo, [2Fe–2S] centres, and a flavin adenine dinucleotide (FAD), all arranged along the intramolecular electron-transfer pathway. In MoCo, the Mo atom is coordinated by the dithiolene sulphurs of a pyranopterin/molybdopterin ligand. During catalysis, the oxygen atom incorporated into hypoxanthine or xanthine is derived from water rather than from molecular oxygen. Substrate hydroxylation is coupled to the two-electron reduction in the molybdenum centre from Mo(+VI) to Mo(+IV), followed by electron transfer from the molybdenum centre through the Fe–S clusters to FAD. Depending on the oxidase/dehydrogenase form, the reducing equivalents are ultimately transferred to molecular oxygen or to NAD+ [8,9,10]. The choice of final electron acceptor depends on the form of XO: the oxidase form generates superoxides and uses molecular oxygen, whereas the dehydrogenase form utilises NAD+ without producing superoxides [11,12]. Although AO has a molecular structure homologous to that of XO and both exhibit similar functions, there are significant differences in substrate specificity [10,13]. Moreover, the sole electron acceptor for AO is molecular oxygen, and AO can oxidise a broader range of substrates than XO [14,15].

Xanthine oxidase is widely distributed across species, highlighting its fundamental role in purine catabolism [8]. In humans, immunohistochemical studies have shown significant XO presence in the epithelial capillaries [16] and substantial amounts in the bile duct parenchyma and liver epithelium [17]. Notably, research by Rosoff et al. has revealed that the liver, among various tissues, contains a significant concentration of Mo [1,2]. This aligns with the broader view that the liver is central to trace-element handling, xenobiotic metabolism, purine catabolism, and redox homeostasis. At the cellular level, Mo homeostasis depends not only on total Mo availability but also on molybdate uptake, MoCo biosynthesis, and the subsequent incorporation of MoCo into active molybdoenzymes. Although molybdate transport has been extensively characterised in prokaryotes and plants, eukaryotic molybdate transport systems remain poorly understood. Nevertheless, a human molybdate transporter (MOT) homologous to algal MOT2 has been described, supporting the concept that regulated molybdate uptake may contribute to intracellular Mo availability and MoCo-dependent enzymatic activity [18,19]. Acquired Mo deficiency is rare in humans but has been reported in the context of prolonged parenteral nutrition. Inherited disturbances of MoCo biosynthesis cause severe MoCD, largely through loss of SOX activity and the accumulation of toxic sulphur-containing metabolites [5,20]. In addition, AO exhibited the greatest activity in numerous organs, including the endocrine, kidney and liver [21].

One of the earliest experiments examining XO activity in the human liver used 189 hepatic tissue samples and found that 21% to 27% of participants showed individual variation in XO activity [22]. Other researchers have reported the liver as one of the XO-rich organs in the human body [23]. However, the clinical approach to XO studies saw a breakthrough in 2002, when Stirpe et al. conducted a survey of XO activity in human hepatic samples. They found that total XO activity was increased in livers with cirrhosis due to viral hepatitis compared with controls. Moreover, they reported that XO activity correlated positively with alanine aminotransferase (ALT) levels, suggesting that XO activity may also correlate with the severity of liver injury [24].

Extensive studies have explored the properties and physiological roles of XO and AO, particularly their relationship with Mo. Numerous investigations involving both animal models [12,25] and human participants have provided valuable insights into XO’s functions [26]. Recent studies have examined XO’s role in liver diseases, including liver physiology in patients with *Plasmodium falciparum* [27] and XO gene expression and activity in individuals with non-alcoholic fatty liver disease (NAFLD) [28]. More recent human studies have further shown that plasma xanthine oxidoreductase (XOR) activity is associated with hepatic steatosis and may reflect liver disease activity across different hepatic pathologies [29,30]. Furthermore, various researchers have reported decreased AO-mediated drug clearance in the cytosols of hepatocytes from individuals with chronic alcohol consumption [31] and have noted that 36 of 239 frequently used medications alter AO activity [32]. Together, these studies indicate that XO/XOR and AO are closely linked to hepatic metabolism and liver pathology. Nevertheless, most available evidence derives from circulating enzyme activity, experimental models, or isolated hepatic drug-metabolism studies, whereas tissue-level relationships between Mo content and molybdoenzyme activity in human liver disease remain poorly characterised. This issue is particularly relevant in end-stage liver failure (ESLF), where advanced parenchymal damage, altered trace-element homeostasis, impaired hepatic metabolism, and the need for liver transplantation may profoundly affect Mo-dependent enzymatic systems [33].

Accordingly, an important area remains unexplored: the correlation between XO activity and Mo content, and between AO activity and Mo content, in liver tissues from allograft donors and ESLF recipients. This study aims to fill this gap by directly assessing hepatic Mo content alongside XO and AO activity in human donor and recipient liver tissue, offering new avenues for research into the roles of XO, AO and Mo-dependent homeostasis in advanced liver pathologies.

## 2. Results

To address the study objective, hepatic Mo content and molybdoenzyme activity in liver tissue samples from liver donors (LD; control group) and recipients with end-stage liver failure. Recipients were further stratified by the primary liver pathology leading to transplantation: hepatitis B or C viral infection (HBV/HCV), alcohol-associated liver disease (ALD), primary sclerosing cholangitis (PSC), Wilson’s disease (WD), and primary biliary cholangitis (PBC). The results are presented below, beginning with XO activity, followed by AO activity, Mo content, and the relationships between these parameters.

### 2.1. Xanthine Oxidase and Aldehyde Oxidase Activity

**Figure 1:**
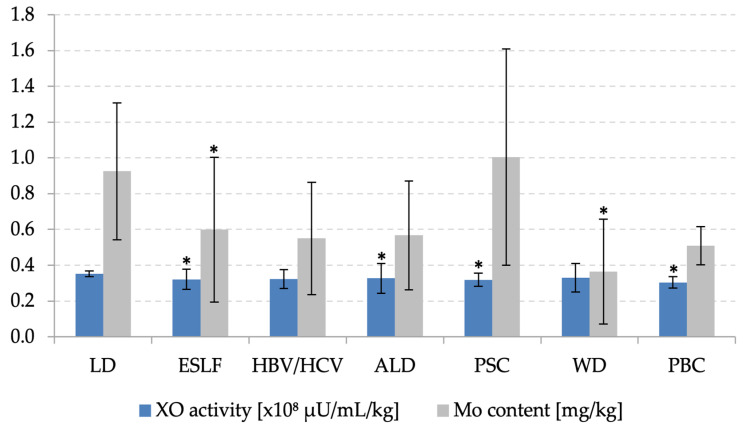
**Xanthine oxidase activity and molybdenum content in liver tissues.** Mean xanthine oxidase (XO) activity [×10^8^ μU/mL/kg] and mean molybdenum (Mo) content [mg/kg] in liver tissue from liver donors (LD; control group), the overall end-stage liver failure (ESLF; study group) group, and ESLF subgroups classified according to the primary liver pathology leading to transplantation. Statistically significant differences compared with the LD group (*p* ≤ 0.05) are marked with an asterisk (*). Overall, XO activity tended to be lower in ESLF subgroups compared with LD controls, whereas Mo content showed greater variability across disease subgroups. Abbreviations: Alcohol-associated liver disease (ALD), hepatitis B or C viral infection (HBV/HCV), primary sclerosing cholangitis (PSC), Wilson’s disease (WD), and primary biliary cholangitis (PBC).

XO activity values for each subcategory are presented in Table 1 and Figure 1. Compared with the LD control group, enzyme activity tended to decrease across all ESLF subgroups. A statistically significant drop in activity was observed in the ALD, PSC, and PBC subgroups. Although the decreases in the HBV/HCV and WD groups were not statistically significant, lower mean XO activity values were also observed in these groups. The highest variability in XO activity was observed in the ALD subgroup, whereas the LD group showed the lowest variability. The mean XO activity for the entire ESLF cohort was 0.321 [×10^8^ μU/mL/kg], compared with 0.352 [×10^8^ μU/mL/kg] in the LD control group. The highest variability was observed in the ALD group at 0.0835, while the smallest was in the LD control group at 0.0165.

Because AO activity [mU/mL] was assessed in the subset of samples with sufficient remaining tissue, the analysis included 25 participants: 8 controls and 17 ESLF recipients. The values in Table 1 were converted to U/mL by multiplying by 10^−3^. In this limited subset, AO activity did not differ significantly between the LD group and the analysed ESLF subgroups.

### 2.2. Molybdenum Content

Molybdenum content in liver tissue is presented in Table 1 and Figure 1. The mean Mo content in the ESLF group (0.599 mg/kg) was significantly lower than that in the control group (0.925 mg/kg; *p* ≤ 0.05), representing an approximately 35% decrease. Among the analysed subgroups, PSC had the highest Mo content (1.004 mg/kg), slightly exceeding the LD population (0.925 mg/kg). However, this difference was not statistically significant. A statistically significant reduction in Mo content was observed in the WD subgroup. Although the HBV/HCV and PBC subgroups showed lower mean Mo content than controls, these differences did not reach statistical significance (*p* = 0.058) and should therefore be interpreted as descriptive trends rather than confirmed group-level effects.

### 2.3. Comparison of Xanthine Oxidase Activity and Molybdenum Content

**Figure 2:**
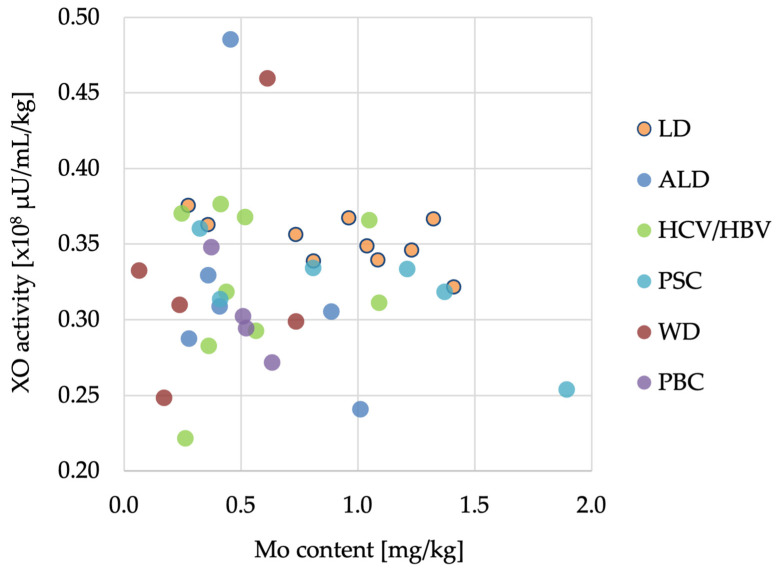
**Distribution of individual XO activity and molybdenum content in liver tissue.** A scatter plot shows individual measurements of xanthine oxidase (XO) activity [×10^8^ µU/mL/kg] and molybdenum (Mo) content [mg/kg] in liver tissue samples from liver donors (LD; control group) and recipients with end-stage liver failure (ESLF), classified by the primary liver pathology leading to transplantation: alcohol-associated liver disease (ALD), hepatitis B or C viral infection (HBV/HCV), primary sclerosing cholangitis (PSC), Wilson’s disease (WD), and primary biliary cholangitis (PBC). Each point represents one participant. The plot illustrates inter-individual variability in hepatic XO activity and Mo content across the analysed groups, with ESLF recipients showing a broader distribution of Mo content than LD controls.

**Figure 3:**
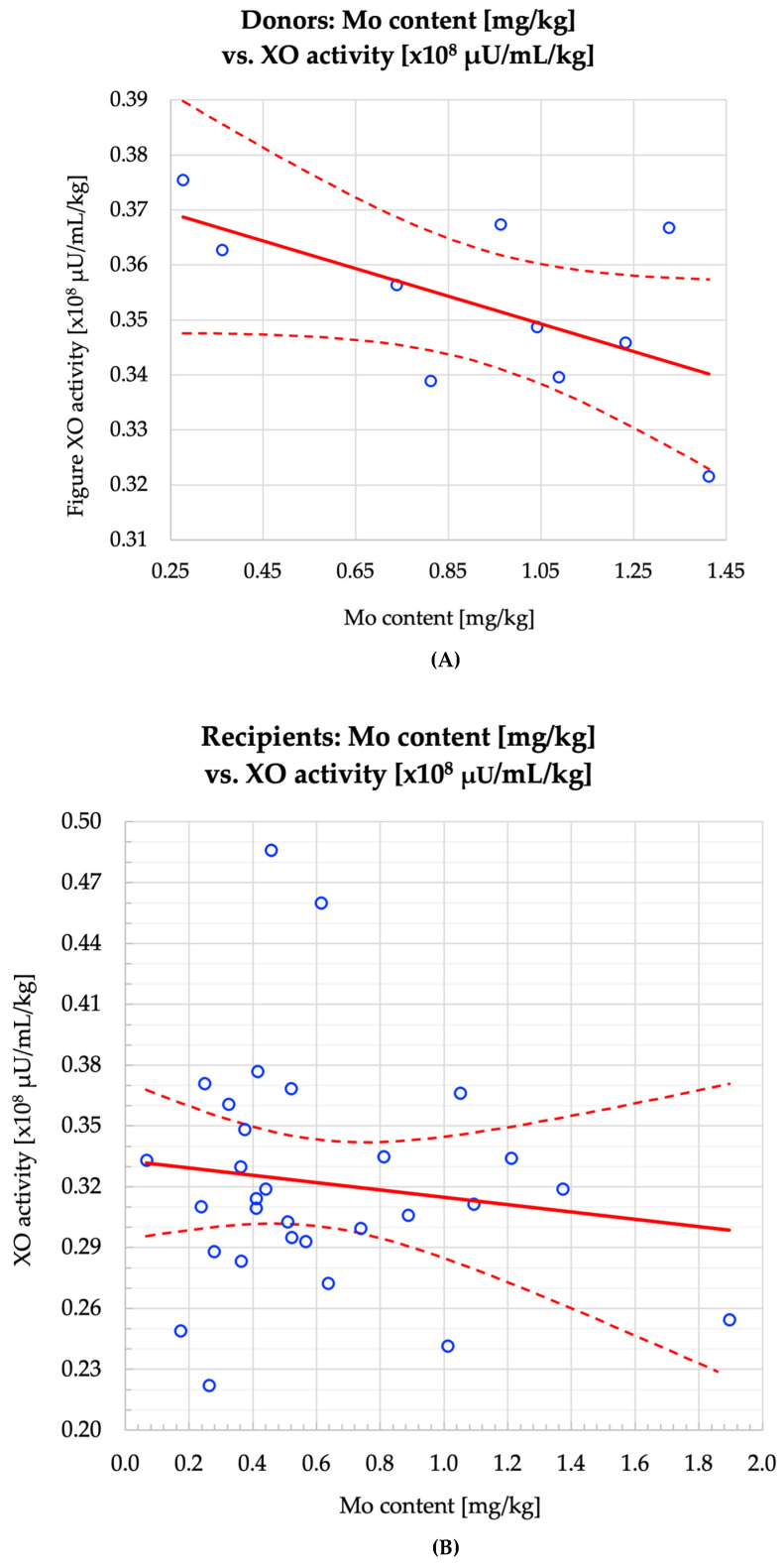
**Pearson correlation between molybdenum content and xanthine oxidase activity in liver tissue.** The scatter plots show the relationship between hepatic molybdenum (Mo) content [mg/kg] and xanthine oxidase (XO) activity [×10^8^ µU/mL/kg] in (**A**) liver donors (LD; control group) and (**B**) liver recipients with end-stage liver failure (ESLF; study group). The solid red line represents the fitted linear regression, while the dashed red lines indicate the 95% confidence bands for the fitted regression line, reflecting the uncertainty of the estimated association. Pearson correlation analysis showed a negative, statistically non-significant association in LD controls (r = −0.5855; *p* > 0.05) and a weak negative, statistically non-significant association in ESLF recipients (r = −0.1297; *p* > 0.05). Overall, the plots indicate a non-significant inverse association between hepatic Mo content and XO activity, with no evidence of a statistically significant correlation in either group. Spearman’s rank correlation was additionally used as a nonparametric sensitivity analysis and supported the absence of a statistically significant monotonic association between Mo content and XO activity in both groups. Abbreviations: Alcohol-associated liver disease (ALD), hepatitis B or C viral infection (HBV/HCV), primary sclerosing cholangitis (PSC), Wilson’s disease (WD), and primary biliary cholangitis (PBC).

Table 1 and Figure 1 compare XO activity and Mo content in liver tissue among the LD group, the whole study group (ESLF), and the individual ESLF subgroups. In general, XO activity was lower in the ESLF than in the LD group, whereas Mo content showed greater variability across the analysed subgroups. These findings indicate that Mo content and XO activity did not change in a strictly parallel manner across all groups. Following the experiments, we investigated the correlation between XO activity and Mo content. To this end, correlation analysis was performed using individual sample values. Pearson correlation analysis was performed separately for LD controls and ESLF recipients, as presented in Figure 2 and Figure 3.

As expected, the donor group exhibited the highest XO activity. However, contrary to our expectations, this group had the second-highest Mo content, surpassed only by the PSC patients. Across the ESLF study group, XO activity generally decreased compared with the control group.

In the control group, a moderate negative correlation between XO activity and Mo content was observed (r = −0.5855, Figure 3A). In contrast, no meaningful correlation was observed in the study group (r = −0.1297; Figure 3B). Interestingly, the PSC subgroup had the highest mean Mo content across all analysed groups, slightly exceeding the control value, despite reduced XO activity in this subgroup. Apart from PSC, the mean Mo content in ESLF subgroups ranged from approximately 0.37 mg/kg to 0.57 mg/kg, markedly lower than the mean in the control group (approximately 0.93 mg/kg). However, only patients in the ALD, HBV/HCV, and WD subgroups exhibited similar XO activity and Mo content. Patients with PBC had similar XO activity but markedly lower mean Mo content than the ALD, HBV/HCV, and WD subgroups. In addition, a nonparametric Spearman rank correlation analysis was performed as a sensitivity analysis and confirmed the absence of a statistically significant association between Mo content and XO activity in both groups. In LD controls, Spearman analysis showed a negative but non-significant association (ρ = −0.5152; *p* > 0.05), whereas in ESLF recipients, no monotonic association was observed (ρ = −0.0122; *p* > 0.05).

## 3. Discussion

In this study, xanthine oxidase (XO) activity was generally lower in patients with end-stage liver failure (ESLF) than in the liver donors (LD; control group). Statistically significant decreases were observed in the ALD, PSC, and PBC subgroups, whereas the HBV/HCV and WD groups showed lower XO activity that did not reach statistical significance. The mean XO activity in liver tissue from the ESLF study group was 0.321 [×10^8^ µU/mL/kg]. The greatest variability (SD) was observed in the ALD subgroup, whereas the lowest variability was found in the control group (LD). Therefore, these results should be interpreted as an overall tendency toward reduced hepatic XO activity in ESLF, rather than as a uniformly significant decline across all disease subgroups.

Regarding Mo content, most ESLF subgroups had lower mean Mo levels than the LD group. A statistically significant decrease was observed only in the WD subgroup. In contrast, the PSC subgroup displayed the highest mean Mo content among all analysed groups, slightly exceeding that of the LD group despite reduced XO activity; however, this difference did not reach statistical significance and was accompanied by the greatest variability (SD). This finding should be interpreted with caution, as the PSC subgroup was small and exhibited high dispersion in Mo values. In addition, cholestatic liver diseases may be associated with marked tissue heterogeneity, biliary remodelling, fibrosis, inflammation, and uneven distribution of parenchymal and non-parenchymal compartments within the sampled tissue. Therefore, the higher mean Mo content in PSC may reflect disease-specific tissue composition or sampling variability rather than preserved or increased Mo-dependent enzymatic function. Across the ESLF cohort, mean Mo content was significantly reduced (35%) compared with the donor control group (Table 1), indicating an overall decrease in hepatic molybdenum levels in patients with ESLF.

The relationship between XO activity and Mo content appeared to differ between the control and ESLF groups. In the LD group, a moderate negative correlation between XO activity and Mo content was observed, whereas no meaningful correlation was detected in the ESLF cohort.

The absence of a direct positive correlation between hepatic Mo content and XO activity may seem unexpected, as Mo is required for the catalytic centre of XO. However, total Mo content measured in liver tissue does not necessarily reflect the fraction of Mo that is biologically available, transported into cells as molybdate, incorporated into MoCo, inserted into mature XO, or retained within catalytically active enzyme. Cellular Mo availability and XO activity may depend on molybdate transport, intracellular trafficking, MoCo biosynthesis, MoCo insertion into apoenzymes, apoenzyme expression, post-translational conversion between xanthine dehydrogenase and oxidase forms, oxidative or inflammatory enzyme modification, and the stability of mature molybdoenzymes. Therefore, changes in total tissue Mo may be uncoupled from XO activity if Mo uptake, storage, export, MoCo assembly, or enzyme maturation is altered. In ESLF, advanced fibrosis, cirrhotic remodelling, cholestasis, inflammation, altered membrane transport, disrupted intracellular molybdate handling, and resultant hepatocyte loss may therefore weaken or abolish the relationship between hepatic Mo concentration and measurable XO activity [2]. This interpretation is also consistent with previous trace-element work on transplant-derived liver tissue, which shows that cirrhotic recipient livers may exhibit disease-context-dependent metal accumulation and co-accumulation patterns [34]. Taken together, this may explain why Mo content and XO activity did not change in parallel across all subgroups and why the ESLF cohort showed no meaningful correlation between these parameters.

Further support for this transport-related concept comes from studies of eukaryotic molybdate transport systems. In *Chlamydomonas reinhardtii*, MOT1 was identified as a high-affinity molybdate transporter, establishing one of the earliest eukaryotic models of regulated molybdate uptake [35]. MOT2 was later described as a distinct molybdate transporter family shared by algae and humans [18]. More recently, insertional mutagenesis in *C. reinhardtii* identified two candidate ABC-type transport proteins, Cre05.g234400 and Cre03.g191350, as potential additional regulators of Mo uptake/storage and Mo efflux, respectively [36]. Although these findings were obtained primarily in microalgae and cannot be directly extrapolated to human liver tissue, they emphasise that Mo homeostasis is regulated at multiple levels of transport and trafficking, not merely by total elemental abundance. This broader framework may help explain why total hepatic Mo content did not correlate with XO activity in ESLF.

Stirpe’s conclusion regarding XO activity differs notably from our findings, as our patients with virus-related cirrhosis (HBV/HCV subgroup) had lower XO activity than liver-healthy controls. However, this comparison should be interpreted with caution because Stirpe et al. analysed total XOR activity, xanthine dehydrogenase (XDH) activity, and XO activity separately, whereas our study focused on XO activity relative to hepatic Mo content. In their study, the main significant finding was an increase in total XOR activity in virus-related cirrhosis, while neither XO activity nor the percentage of XO activity differed significantly between the analysed groups [24]. Therefore, our lower XO activity in the HBV/HCV subgroup does not necessarily contradict Stirpe’s findings but may reflect a different enzymatic component of the XOR system.

Several additional factors may explain the discrepancy between the two studies. Stirpe et al. used histologically normal liver tissue adjacent to extrahepatic metastases as the control group, whereas our control group comprised liver donors without diagnosed liver pathology. Moreover, their virus-related cirrhosis group was substantially larger and was analysed as a combined HBV/HCV cohort, whereas our HBV/HCV subgroup was smaller and comprised patients with ESLF requiring transplantation. Importantly, Stirpe et al. reported that total XOR activity correlated positively with ALT, suggesting a relationship with active hepatocellular injury or viral replication, whereas our study assessed explanted end-stage liver tissue, in which advanced fibrosis, architectural remodelling, and hepatocyte loss may reduce measurable parenchymal XO activity.

Stirpe et al. also reported that the percentage of XO activity correlated with AST, bilirubin, partial thromboplastin time, prothrombin time, and warm ischaemia, suggesting that the XO/XDH balance may be influenced by liver impairment, tissue injury, and perioperative or pre-analytical factors [24]. These variables were not uniformly available in our cohort and, therefore, could not be included in the present analysis. Consequently, the divergence between Stirpe’s findings and our results is likely multifactorial and may relate to differences in the measured enzymatic parameter, control tissue selection, disease stage, subgroup size, clinical covariates, and tissue sampling conditions.

Currently, most available data on XO activity are derived from studies measuring XO activity in serum [26,27,29,37,38], whereas only a handful of authors have explored the functions of XO [22,23,24,39] and AO [31,39,40] in the liver parenchyma, and even fewer have addressed the topic from a clinical perspective.

Although our research focused on liver parenchymal XO activity and its relationship with hepatic Mo content, serum-based studies remain important for interpreting the broader clinical relevance of XO in liver disease. Therefore, to provide a comprehensive discussion of the dissertation’s topic, we briefly summarise the importance of XO as established by these studies. Some researchers report that increased serum XO activity is a biochemical marker of *Plasmodium falciparum*-associated liver failure [27], while others link elevated serum XO activity to the development of NAFLD [28]. This connection is particularly important, as NAFLD is the most common liver pathology globally and a leading cause of liver-related morbidity [41]. Moreover, the relationship among NAFLD, XO activity, and hyperuricemia is well established. Xu et al. studied XO activity and expression in both cellular and mouse models, finding that XO levels increased in NAFLD and that knocking down XO expression notably reduced uric acid production [28]. Additionally, hyperuricemia has been shown to significantly increase the risk of NAFLD, suggesting that uric acid or XO itself may be a therapeutic target for managing NAFLD. In our cohort, hepatic XO activity was generally reduced, suggesting that the elevation in serum XO activity reported by other researchers may not directly reflect tissue XO activity in end-stage liver tissue. Instead, circulating XO/XOR activity may reflect enzyme release, systemic inflammation, endothelial involvement, or active hepatocellular injury rather than preserved enzymatic activity within explanted cirrhotic parenchyma.

Another study of serum XO levels in 64 patients with chronic liver disease and 12 controls found that, in patients with chronic liver disease, particularly those with cholestasis, serum XO levels were markedly elevated compared with controls, consistent with prior reports. However, no correlation was observed between XO levels and histological markers of hepatocyte necrosis [26]. This further supports the view that elevated circulating XO may reflect cholestasis-related, inflammatory, or systemic processes rather than directly reflecting parenchymal XO activity.

In the context of our findings, it is important to emphasise that molybdenum is the only second-row transition metal essential for most organisms, serving as a cofactor in several key enzymes, including XO, AO, SOX, and mARC [42,43,44]. These molybdenum-dependent enzymes have been extensively reviewed [2,10,20,45,46] and are vital for maintaining metabolic homeostasis and facilitating redox reactions involved in processes such as purine oxidation, sulphur amino acid metabolism, and aromatic compound breakdown [3,47,48,49]. The liver, as the primary Mo storage organ, is particularly susceptible to Mo-related changes, and elevated Mo levels can induce hepatotoxicity through mechanisms such as oxidative stress and mitochondrial damage [50]. Molybdenum distribution varies significantly across human tissues, with the liver showing the highest content (1.1 mg/kg) [51], which aligns with our finding of 0.9 mg/kg in the LD group. Due to the limited availability of human liver samples and the insufficient sensitivity of many analytical techniques, directly comparable results in other studies are scarce.

Primary Mo deficiency is rare, although genetic defects in MoCo synthesis can lead to MoCD [52,53]. Elevated Mo levels have been detected in the serum of patients with other liver-related conditions, including seropositive chronic viral hepatitis, liver cirrhosis, alcohol-associated liver disease, liver metastases, gallstones, gallbladder and bile duct tumours, and pancreatic carcinoma. Additionally, elevated Mo was found in cases of primary biliary cirrhosis and some drug-induced liver injuries [53]. On the contrary, the co-exposure to Mo and cadmium was reported to activate the cyclic GMP-AMP synthase-stimulator of interferon genes (cGAS-STING) signalling pathway to inhibit the Sam50-MICOS-ATAD3 (mitochondrial membrane proteins) axis. This mechanism reduces mitochondrial DNA (mtDNA) stability and promotes hepatic inflammatory responses in sheep, including increases in TNF-α, CRP, and IL-18 levels [54]. However, Mo accumulation was not observed in our cohort with primary ESLF. Therefore, our data do not support a pattern of marked hepatic Mo accumulation in this cohort. Nevertheless, because environmental exposure, dietary intake, occupational history, renal elimination, and longitudinal Mo kinetics were not assessed, the contribution of external Mo exposure to liver pathology cannot be definitively excluded. Overall, these findings suggest that marked Mo accumulation was not a dominant feature of the analysed ESLF tissue samples.

### 3.1. Limitations

Our study encountered several challenges, the most significant of which were the limited availability of biological material and small tissue sample sizes. Given the unpredictable nature of liver transplantation procedures, it was impossible to predetermine the number of subgroups or accurately forecast their sizes. This inherent uncertainty made it difficult to ensure balanced group sizes and sufficient statistical power.

Another important limitation is the lack of adjustment for demographic and clinical variables, including age, sex, disease duration, fibrosis stage, cholestasis parameters, inflammatory markers, renal function, and nutritional status. In particular, clinical indices such as MELD score, Child–Pugh class, bilirubin, ALT, AST, GGTP, INR, albumin, and creatinine could influence trace-element status or enzymatic activity [24,55]. Because these variables were not uniformly available for all participants or were not included in the current analysis, we could not determine whether the observed differences in Mo content, XO activity, or AO activity were independently associated with ESLF status or were partly influenced by clinical heterogeneity. Future studies should integrate biochemical, histological, and demographic data to allow multivariable adjustment and disease-severity stratification.

Moreover, a substantial proportion of our study population comprised individuals with rare liver diseases, such as PSC, PBC, and WD [56,57], further narrowing the pool of eligible participants and exacerbating recruitment challenges. The rarity of these conditions limited the diversity and size of our study cohort, potentially affecting the generalisability of the findings. Furthermore, had the study group been larger, some currently statistically insignificant differences might have crossed the barrier of statistical significance (*p* ≤ 0.05) and been estimated with greater precision.

### 3.2. Strengths

Despite the limitations noted above, our study has several important strengths. To our knowledge, it is the first to investigate and compare Mo content with both XO and AO activity in patients with various underlying causes of ESLF. This pioneering approach offers valuable insights into the biochemical alterations associated with liver disease, particularly in rare conditions such as PSC, PBC, and WD.

Additionally, selecting a well-defined control group of LDs without diagnosed hepatic pathology provides a reliable baseline for comparison. The rigorous inclusion criteria and subsequent review of participant medical records further enhance the study’s credibility, as any confounding factors, such as drug abuse or unidentified toxic liver injury (excluding alcohol), are carefully identified and excluded. This attention to detail enhances the accuracy and validity of the study’s results, providing a valuable foundation for generating new hypotheses in the fields of metallomics and hepatology.

### 3.3. Future Directions

We believe that further studies of hepatic redox enzymes are needed, ideally involving larger, more diverse populations. Expanding the sample size in future research would enhance statistical power and provide a clearer understanding of enzyme behaviour across different liver disease groups, thereby strengthening overall conclusions. By gaining deeper insights into the role of molybdenum-dependent flavoproteins in human liver diseases, we could potentially uncover new diagnostic procedures and treatment approaches, leading to advancements in both patient care and medical understanding. Therefore, we suggest a few new hypotheses worth pursuing.

Firstly, recent data indicate that increased urinary Mo excretion correlates with beneficial (anti-inflammatory) effects in the liver [58]. It remains unclear whether higher elimination reflects hepatic Mo accumulation (a net surplus) or enhanced clearance that prevents hepatic accumulation.

Secondly, a study examining changes in Mo concentration during the development of N-nitrosodimethylamine (NDMA)-induced hepatic fibrosis reported significant alterations. In the second and third weeks following NDMA administration, serum Mo levels rose markedly, whereas liver Mo content declined, reaching a maximum reduction at three weeks. A strong negative correlation was observed between increased serum and decreased liver Mo concentrations in rats, suggesting that in NDMA-induced liver fibrosis, Mo may be mobilised from the liver into the bloodstream [59]. Interactions of Mo with other elements, particularly iron and zinc (Zn), may contribute to this mobilisation. Additionally, with Zn acting as a cofactor for superoxide dismutase, an antioxidant enzyme, Mo-Zn interactions may yield particularly impactful effects in ESLF pathogenesis.

Thirdly, the observed inverse tendency between Mo content and XO activity is intriguing, particularly because Mo is required for the catalytic centre of XO. Although this relationship did not reach statistical significance and should therefore be interpreted cautiously, it raises the possibility that total hepatic Mo content may not directly reflect Mo bioavailability, MoCo incorporation, or mature XO activity. This finding is also notable because MoCo biology may extend beyond XO and AO activity alone. Elevated expression of MoCo synthesis 2 enzymes has been associated with lower glucose levels and modest β-cell regeneration [60]. Clarifying how altered Mo availability or MoCo-dependent metabolism affects these pathways would be valuable, particularly given that neonatal MoCD leads to severe consequences, including epileptic encephalopathy, hypotonia, failure to thrive, and respiratory failure [52].

## 4. Materials and Methods

### 4.1. Participants

Our study primarily included 42 participants, divided into two major cohorts: a control group (*n* = 10) and a study group (*n* = 32). The control group comprised liver donors (LDs) with no diagnosed liver pathology, making their organs suitable for transplantation. The study group comprised liver recipients diagnosed with liver pathology resulting in end-stage liver failure (ESLF) who required liver transplantation (LTx). The ESLF group was further subdivided by the primary liver pathology diagnoses leading to LTx, with the following subgroups: (1) hepatitis B or C viral infection (HBV/HCV), (2) alcohol-associated liver disease (ALD), (3) primary sclerosing cholangitis (PSC), (4) Wilson’s disease (WD), and (5) primary biliary cholangitis (PBC).

In addition, following a review of medical records, two patients were excluded from the study–one from the HBV/HCV subgroup and one from the WD subgroup. One had a history of multisubstance drug abuse, while the other presented with an unidentified toxic liver injury superimposed on primary liver pathology. Therefore, the final study population comprised 40 participants, with these two excluded to avoid confounding.

Liver tissue samples were collected from each participant during the ‘back table’ step of the surgical procedure, i.e., after hepatectomy and prior to transplantation, at the same time point for each LTx. Sample collection followed standard surgical protocols and was approved by the Bioethics Committee of the Medical University of Warsaw (KB/146/2006). Each sample was collected under sterile conditions from the 5th and 6th liver segments, packed into individually coded containers, and stored at −80 °C. Before each experimental procedure, including the determination of XO activity and Mo concentration, the frozen tissues were thawed, sterilely divided into smaller pieces, and weighed.

After performing XO activity measurements, we had remaining, unused tissue samples from 8 donors (controls) and 17 liver allograft recipients. Therefore, we decided to determine whether AO activity would be altered in this patient cohort. Aldehyde oxidase is a xanthine oxidase-like molybdenoflavoenzyme [10]. The composition of the subgroups and the results are presented in Table 1.

### 4.2. Determination of the Activity of Xanthine Oxidase and Aldehyde Oxidase

The study used frozen tissue samples. The first stage of enzyme activity analysis involved mechanical homogenisation of frozen tissues using the TissueLyser device (Qiagen, Hilden, Germany). Enzyme activity was measured with a Perkin-Elmer LS-50B spectrofluorometer (PerkinElmer, Waltham, MA, USA). The analysis was conducted using the Xanthine Oxidase Fluorometric Assay Kit (Cayman Chemical, Ann Arbor, MI, USA) and Aldehyde Oxidase Assay Kit (Creative BioMart, Shirley, NY, USA), both according to the manufacturer’s protocols. The test was performed in duplicate.

### 4.3. Molybdenum Content Analysis

The analytical procedure was designed based on previous experience with elemental analysis of small biological samples [61]. Molybdenum content in lyophilised liver samples (0.5 mg–30 mg dry-weight) was determined using inductively coupled plasma mass spectrometry (ICP-MS). Prior to analysis, liver tissues were mineralised in concentrated nitric acid (65% HNO_3_, suprapure grade) using a microwave digestion system (UltraWAVE, Bergamo, Italy). Due to the limited availability of biological material, very small sample masses were placed in PTFE digestion vessels and digested using a protocol that reached 200 °C. The acid volume was adjusted proportionally to the sample mass (approximately 0.5 mL). The digests were diluted with Milli-Q water to achieve a final acid concentration of no more than 5%.

Molybdenum concentrations were determined using an ICP-MS spectrometer (NexION 300D, PerkinElmer, Waltham, MA, USA). Two isotopes (^97^Mo and ^98^Mo) were monitored, yielding consistent results. Quantification was performed using an external calibration curve covering a concentration range from 0.5 μg/L to 50 μg/L. Each digest was analysed in triplicate, and the relative standard deviation did not exceed 3%.

Limits of detection (LOD) and quantitation (LOQ) were calculated based on 10 measurements of procedural blanks under the same conditions as the samples and were defined as 3 and 10 times the standard deviation of the blank signal, respectively. The obtained values were converted to concentration units (mg/kg), accounting for the average sample mass used in the analysis. The resulting LOD and LOQ values were 0.12 mg/kg and 0.14 mg/kg, respectively.

The analytical procedure was validated using the certified reference material (CRM) NIST SRM 1577c (bovine liver, NIST, Gaithersburg, MD, USA). The measured Mo content corresponded to a recovery of approximately 95%, even when CRM subsamples approximately ten times smaller than the minimum recommended sample mass for elemental analysis (100 mg) were analysed. Results are reported on a dry-weight basis.

### 4.4. Statistics

Statistical analyses were conducted using STATISTICA 13.1 and Microsoft Excel (version 16.109.1). XO and AO activity data were reported as means and standard deviations. Because of the limited and unequal sample sizes across the analysed groups, and the exploratory nature of the subgroup comparisons, between-group differences were assessed using the nonparametric Mann–Whitney U test, which does not require the assumption of normal distribution. Statistical significance was set at *p* ≤ 0.05.

Correlations between pairs of continuous variables were assessed using Pearson’s correlation coefficient. As the parameters were continuous, the aim was to assess the strength and direction of potential linear association. Scatter plots were visually inspected to evaluate the direction and approximate linearity of the relationship between Mo content and XO activity. However, due to the small sample size, particularly in the LD group, normality assumptions could not be robustly verified; therefore, the correlation results should be interpreted as exploratory and hypothesis-generating rather than confirmatory. A nonparametric Spearman rank correlation analysis was also performed as a sensitivity analysis. Due to the limited number of patients in individual disease subgroups, the results should be interpreted with caution.

## 5. Conclusions

Our study demonstrated a significant decrease in both xanthine oxidase (XO) activity and molybdenum (Mo) content in liver tissue samples from patients with end-stage liver failure (ESLF) undergoing liver transplantation, compared with liver donors (healthy controls). At the subgroup level, XO activity was significantly reduced in alcohol-associated liver disease (ALD), PSC, and PBC, whereas HBV/HCV and Wilson’s disease showed lower but non-significant values. Mo content was significantly reduced in the overall ESLF cohort and in the Wilson’s disease subgroup, although disease-specific variability was observed, including relatively high Mo content in the PSC subgroup. No significant differences were observed in aldehyde oxidase activity between donor and recipient liver tissues in the analysed subset. These findings suggest that ESLF is associated with disturbances in hepatic elemental composition, including Mo, and with altered Mo-dependent enzymatic activity. To better define hepatic metabolism and the biological effects of trace elements, future studies should examine liver tissue alongside corresponding serum and portal blood samples.

## Figures and Tables

**Table 1:** **Comparison of xanthine oxidase (XO) activity [×10^8^ μU/mL/kg], molybdenum (Mo) content [mg/kg], and aldehyde oxidase (AO) activity [U/mL] in the liver donors (LD; control group), study subgroups, and the whole study group (ESLF).** The values are presented as mean and standard deviation (SD). The *p*-values represent comparisons with the control group (Mann–Whitney U test). Abbreviations: Alcohol-associated liver disease (ALD), hepatitis B or C viral infection (HBV/HCV), primary sclerosing cholangitis (PSC), Wilson’s disease (WD), and primary biliary cholangitis (PBC).

GROUP	*n*	XO Activity			Mo Content			*n*	AO Activity		
		Mean	SD	*p*	Mean	SD	*p*		Mean	SD	*p*
		[×10^8^ µU/mL/kg]			[mg/kg]				[U/mL]		
**LD**	10	0.352	0.0165	–	0.925	0.383	–	8	8.805	4.714	–
**HBV/HCV**	9	0.323	0.0523	0.438	0.550	0.313	0.058	–	–	–	–
**ALD**	6	0.327	0.0835	**0.045**	0.567	0.304	0.116	6	15.095	11.007	0.518
**PSC**	6	0.319	0.0358	**0.020**	1.004	0.604	0.871	6	6.614	2.480	0.651
**WD**	5	0.330	0.0788	0.098	0.365	0.293	**0.011**	5	14.126	8.450	0.478
**PBC**	4	0.304	0.0319	**0.028**	0.510	0.107	0.058	–	–	–	–
**ESLF** **(study)**	30	0.321	0.0566	**0.012**	0.599	0.405	**0.035**	17	11.817	8.572	0.522

## Data Availability

The original contributions presented in this study are included in the article/Supplementary Material. Further inquiries can be directed to the corresponding authors.

## Supplementary Materials

The following supporting information can be downloaded at: https://www.mdpi.com/article/10.3390/ijms27114998/s1.

## Abbreviations

The following abbreviations are used in this manuscript:

Mo	Molybdenum
MoCo	Molybdenum Cofactor
MPT	molybdopterin
MoCD	Molybdenum Cofactor Deficiency
XO	Xanthine Oxidase
AO	Aldehyde Oxidase
XOR	Xanthine Oxidoreductase
XDH	Xanthine Dehydrogenase
SOX	Sulphite Oxidase
mARC	Mitochondrial Amidoxime-reducing Component
MOT	Molybdate Transporter
mtDNA	Mitochondrial DNA
FAD	Flavin Adenine Dinucleotide
ESLF	End-stage Liver Failure
LD	Liver Donor
NAFLD	Non-alcoholic Fatty Liver Disease
HBV/HCV	Hepatitis Type B/C
PSC	Primary Sclerosing Cholangitis
PBC	Primary Biliary Cholangitis
WD	Wilson Disease
ALD	Alcohol-associated Liver Disease
LTx	Liver Transplantation
ALT	Alanine Aminotransferase
